# The Past and Present in Dentistry

**Published:** 1898-04

**Authors:** C. N. Peirce

**Affiliations:** Philadelphia


### THE PAST AND PRESENT IN DENTISTRY.

BY C. N. PEIRCE, D.D.S., PHILADELPHIA.

It has been stated that the evolution of languages, regarded as a psychological process, is largely determined by social conditions.

The following inquiry seems pertinent: Is there a profession, an industry, an enterprise of any kind affecting the human family that has been so shackled, so embarrassed, so handicapped by social conditions as the profession of dentistry? Compare its position with that of the obstetrician, the opthalmologist, the laryngologist, or, indeed, any specialty of medicine; socially it is in the shade. Is the limited education possessed by the masses in the profession an important factor in this? Or is it the occupation itself which places a stigma upon the character of a dentist regardless of other conditions? It has been said by a prominent dentist that within the limits of the dental profession there are men so skilled in medicine and the arts that, were those pursuing the latter stricken with paralysis and the former disabled by accident, their ranks could be so quickly filled by members of the dental profession that the ripple upon the wave of society would be but temporary indeed. However this may be, there are those in the profession whose intelligence and education should be a passport admitting wherever they desired, without question or comment. Believing in evolutionary processes, and that dental education in its development is not unlike other growths, its votaries have been persistent in their efforts to advance its status from the simple to the complex, from the elementary to the scientific; yet there have been periods in this progressive stage that may be denominated as expressions of degeneracy. Of these we will speak later on.

A hasty glance at the condition of dentistry and dental education fifty or seventy-five years ago, and a comparison with its condition forty or more years later, or at a more recent period, will demonstrate the marvellous change.

In early times an applicant for instruction was charged a fee, the amount of which was in an inverse ratio to the time to be spent in obtaining the desired information. Six hundred dollars for three months, or two hundred for twelve, was not an unusual fee demanded and paid.

The value to the prospective dentist of this economy in time was almost without price, for in a few months his fortune was supposed to be secured. Nor could the preceptor be more jealous and careful regarding the character of the one to whom he was intrusting the secrets of his laboratory and admitting to his office to watch the movements of both patient and operator. The result of this care brought men of character into the profession.

In 1846, when the first dental college opened for the reception of students, it was regarded with but little favor by those whose annual income had been materially augmented by fees given for private instruction. In order that the college should enjoy the patronage of a sufficient number of students to pay interest on the outlay and necessary expenses, it was quite incumbent on those conducting the enterprise that very limited requirements in anatomy, physiology, and chemistry should be exacted, and also limit in time required to obtain a degree and an attesting parchment. But, limited as it was, it was an advance in the opportunity offered for thorough, honest teaching in the practical branches,—operative and prosthetic. One important element, however, was eliminated,—the personal contact with and the influence of teacher upon pupil.

Within six years from the establishment of the first school, with fifty students in attendance, three were in successful operation, with a patronage of a little more than two hundred students in the three. In 1870 five schools had been established, their combined classes numbering but about, or less than, three hundred students, while in 1896 the country enjoyed the disgrace, shall I say, of fifty-eight dental schools, with over six thousand five hundred (6500) names on their matriculating lists.

That one or more schools with centres and agents for the sale and distribution of diplomas have been established in this country is a well-recognized fact, but it must be remembered that these have only been in response to the demand for a parchment giving the holder thereof the right to practise dentistry. Hundreds of these unearned documents have been distributed over this as well as the various countries of Europe, thereby increasing the number of charlatans in the profession, and helping not a little to sink it into that slough of despair and degradation in which it is now involved.

It was the activity of this villanous practice that led the dental profession to seek regeneration and protection in State examining boards. These were to be the "antitoxin,"—the source of redemption; much was anticipated, hoped for, by many sincere and able men in the profession. Before making the inquiry as to the extent of the remedy secured from these examining boards and the realization of the hopes of their originators, let me pay a well-merited tribute to the integrity, honesty, and singleness of purpose of the professors and teachers conducting many of our schools. Men more devoted, more earnest in their advocacy of, and in their efforts for, the advancement of the dental profession are not to be found. This is well illustrated in the adoption as rapidly as possible of a broader curriculum, an increased length of each session, and an addition to the number of sessions required before a candidate for graduation could be admitted to final examination. This speaks emphatically for such schools, and demonstrates the purpose of the faculties in charge.

In recurring now to the inquiry regarding examining boards: Have they given satisfactory evidence of a due appreciation of their responsible position? Has their influence been for the protection and encouragement of the best schools? Are the men composing the boards as well versed in the science and art of dentistry as the professors in the best schools? Have they given evidence of any better, or as great an interest in the advancement of the profession? Are they men of equal scientific attainments, or of a higher, unswerving moral character? These are pertinent questions. What the profession—the higher element in it—has a right to demand is that competent, conscientious teachers shall be protected against charlatanism in any form, and that proficient students, recent graduates, well educated in all departments, shall be dealt with justly,—shall be relieved of a rivalry with the ignorant and unscrupulous. The truly professional men, and teachers, are discouraged, not to say utterly disheartened, at the commercial spirit which pervades educational institutions to-day. The atmosphere of barter and sale infests the class-room, and high ideals in the pursuit of a chosen profession are not encouragingly present in the majority of students.

In a dental school the recital of a practical case, or the description of a manipulative process, has every ear and eye; not a word or movement to be lost. But the origin of a structure, the etiology of a disease, the evolution of a form,—not having a fee apparent or prospective,—is a matter of inattention and indifference.

Have the examining boards tended to remedy this evil? Have they encouraged the student to ideals? Do they estimate the value of well-grounded principles and urge to proficiency in such? Or, are they not, at least some of them, burdened with the same commercial spirit, seeing no value in anything save possessed of an earning capacity?

The position of a critic is an unpleasant one, but when devoid of personality or malice may stimulate to improved conditions.

A student having completed *only the first year's course in college*, after an examination by a State dental board, received the following certificate:

"It gives me great pleasure to inform you that your examination before the Board was satisfactory in every respect, and I have been instructed to express to you the appreciation of the members of the Examining Board as to the high standard of the work which showed such careful and thorough preparation on your part, and at the same time to congratulate you on the fact of your being the first applicant who has come up under (*the five years' clause*) our present arrangement who has passed the examination at his first examination. We wish you the greatest success in the profession you have entered under such favorable auspices."

(Signed by Secretary of the Examining Board.)

This certificate naturally led the holder to believe it insured entrance into the senior class, and for that purpose was presented to the dean. This application was positively refused, but that the applicant might also be convinced of unfitness for such promotion was asked to submit to an examination in physiology, after which the request was not repeated.

A graduate of more than usual merit, having received a diploma, which was shown to an examining board, passed before said board all examinations satisfactorily, at least was so informed by a member thereof, who also extended personal congratulations, and requested a friend to write a letter to the successful individual "expressing in complimentary terms the satisfaction given the board."

A few days subsequent to the *personal interview* and reception of congratulatory letter, imagine the young man's surprise at receiving the following from the Secretary of the Board:

"Dear Sir,—I regret to inform you that your examination before the Examining Board was not satisfactory, and it will be necessary for you to refrain from practising dentistry in the State. You will have an opportunity for a re-examination."

(Signed by Secretary of the Board.)

(Date in July.)

This correspondence was shown to a man of prominence, an ex-representative, and three days thereafter the applicant received the necessary certificate, and without another examination or waiting four months for it.

These facts are certainly evidence of the absence of any fixed principles, or of a settled policy on the part of the board to advance the status of the profession or of dental education.

Moral. Were personal considerations a factor in the above cases? Evidence of dishonesty or degeneracy are always painful to recall and record, but unless some steps are taken to check this downward tendency and avert its consequences, dental education will be greatly impeded and the position of the profession a travesty, a burlesque, on a scientific pursuit.

